# Biallelic EPCAM deletions induce tissue-specific DNA repair deficiency and cancer predisposition

**DOI:** 10.1038/s41698-024-00537-6

**Published:** 2024-03-11

**Authors:** V. J. Forster, M. Aronson, C. Zhang, J. Chung, S. Sudhaman, M. A. Galati, J. Kelly, L. Negm, A. B. Ercan, L. Stengs, C. Durno, M. Edwards, M. Komosa, L. E. Oldfield, N. M. Nunes, S. Pedersen, J. Wellum, I. Siddiqui, V. Bianchi, B. R. Weil, V. L. Fox, T. J. Pugh, J. Kamihara, U. Tabori

**Affiliations:** 1 The Arthur and Sonia Labatt Brain Tumour Research Centre, The Hospital for Sick Children, Toronto, ON Canada; 2 Zane Cohen Centre, Sinai Health System and Faculty of Molecular Genetics, University of Toronto, Toronto, ON Canada; 3 Division of Gastroenterology, Hepatology and Nutrition, The Hospital for Sick Children, Toronto, ON, Canada; 4 University Health Network, Toronto, ON Canada; 5 Department of Paediatric Laboratory Medicine and Pathobiology, Division of Pathology, The Hospital for Sick Children, Toronto, ON, Canada; 6 Department of Surgery, Boston Children’s Hospital and Harvard Medical School, Boston, MA USA; 7 Department of Pediatric Oncology, Dana-Farber Cancer Institute, Boston, MA USA; 8 Division of Gastroenterology, Hepatology and Nutrition, Boston Children’s Hospital, Boston, MA USA; 9 Division of Haematology/Oncology, The Hospital for Sick Children, Toronto, ON, Canada; 10 Department of Medical Biophysics, University of Toronto, Toronto, ON, Canada

**Keywords:** Paediatric cancer, Cancer genetics

## Abstract

We report a case of Mismatch Repair Deficiency (MMRD) caused by germline homozygous *EPCAM* deletion leading to tissue-specific loss of MSH2. Through the use of patient-derived cells and organoid technologies, we performed stepwise in vitro differentiation of colonic and brain organoids from reprogrammed EPCAM^del^ iPSC derived from patient fibroblasts. Differentiation of iPSC to epithelial-colonic organoids exhibited continuous increased EPCAM expression and hypermethylation of the MSH2 promoter. This was associated with loss of MSH2 expression, increased mutational burden, MMRD signatures and MS-indel accumulation, the hallmarks of MMRD. In contrast, maturation into brain organoids and examination of blood and fibroblasts failed to show similar processes, preserving MMR proficiency. The combined use of iPSC, organoid technologies and functional genomics analyses highlights the potential of cutting-edge cellular and molecular analysis techniques to define processes controlling tumorigenesis and uncovers a new paradigm of tissue-specific MMRD, which affects the clinical management of these patients.

## Introduction

Cancers caused by mismatch repair deficiency (MMRD) share unique characteristics including universal hypermutation, microsatellite instability (MSI), and specific genomic signatures^1,2^. MMRD can occur as a somatic event in many cancer types or in individuals carrying germline pathogenic variants in the MMR genes. Lynch Syndrome (LS) defines individuals harboring heterozygous pathogenic MMR variants in *MSH2*, *MSH6*, *MLH1, PMS2*, or *EPCAM* genes. LS patients are at increased risk of early-onset cancers, mostly colorectal and endometrial^3^. Biallelic pathogenic variants in the mismatch repair genes^4^ result in constitutional mismatch repair deficiency (CMMRD) which is arguably the most aggressive multiorgan cancer predisposition syndrome in humans.

Patients with CMMRD have a complete loss of MMR in all tissues and therefore develop synchronous and metachronous malignancies beginning in early childhood^5^. All organs are affected, however, the most common cancers include brain, gastrointestinal (GI) and hematopoietic malignancies^6^. As a result, survival was extremely poor and most patients with CMMRD did not reach adulthood.

Recently, several functional genomic tools enabled accurate analysis of mutational burden^1,7,8^ and MSI enabled accurate diagnosis of MMR in normal and malignant cells establishing the diagnosis for CMMRD, LS and corresponding cancers.

Furthermore, aggressive surveillance recommendations^9^ were developed and have been shown to dramatically improve survival for individuals with CMMRD^10^. Given the high risk of malignancies occurring across multiple organs, the current CMMRD surveillance protocol involves intensive multimodality and frequent screening from a young age. It is therefore imperative to define which organs are at risk for cancer development in order to design appropriate surveillance protocols and interventions.

*EPCAM*, an epithelial cell-specific adhesion molecule with no known direct function in DNA repair processes has been implicated to cause LS as its gene is located 17 kb upstream of *MSH2*. *EPCAM* 3′ deletions result in ablated EPCAM transcriptional termination, aberrant transcription leading to downstream transcriptional interference and *MSH2* promoter DNA hypermethylation resulting in loss of *MSH2* expression^11^. Patients harbouring heterozygous *EPCAM* deletions are predisposed to GI cancers while involvement of genitourinary cancers^12^ and other LS cancers are rare. Importantly, biallelic germline deletions in *EPCAM* have not been extensively studied and consequently the resulting tumor spectrum and the implications for multimodal surveillance implementation are unknown.

Since *EPCAM* is only expressed in epithelial tissues, its effect on *MSH2* expression may be tissue-specific and limited to those which are epithelial in origin. We hypothesized that individuals with homozygous *EPCAM* deletion will exhibit a unique “tissue-specific CMMRD” and will develop hypermutation, microsatellite instability and cancers restricted to tissues in which *EPCAM* is expressed. If confirmed, this would allow a more tailored approach to cancer screening in these individuals.

Here we demonstrate evidence of tissue-specific CMMRD in a family with two children with homozygous *EPCAM* deletion. Using patient-derived tissues we generated stem cell and organoid models to perform detailed genetic and functional genomic assays to establish the tissue-specific effect of *EPCAM* deletion on MSH2-led DNA MMRD.

## Results

### Case history

The proband, **(EPCAM**^**del**^, Supplementary Fig. 1) initially presented at 4 years of age with blood in the stool, which was suspected as being secondary to constipation. At age 6, abdominal pain and tissue protruding from the rectum prompted a colonoscopy to evaluate for polyps. Colonoscopy subsequently revealed multiple large colonic polyps, with pathology consistent with low-grade and dysplastic adenomas. Due to continued abdominal pain and polyp formation, at age 8 he underwent total proctocolectomy with surgery involving ileal pouch-anal anastomosis.

The patient’s family history was significant for parental consanguinity (first cousins) and a sister with Bardet Biedl syndrome. The sister had four colonic polyps identified when she presented with rectal bleeding at age 2. She had homozygous deletions of *EPCAM*. She passed away due to multiorgan failure. Both parents had LS due to a heterozygous *EPCAM* deletion and had 1–2 polyps on colonoscopy. The patient’s maternal grandfather was diagnosed with colon cancer at age 60 and was confirmed to have a germline *EPCAM* deletion. Germline testing identified a homozygous 3′ UTR deletion in *EPCAM*. Somatic testing of the proctocolectomy polyps revealed MSI high, hypermutator phenotype (>10 mutations/MB), with 17.54 mutations/MB (Fig. 2c). The patient was subsequently closely monitored between age 8 and 11 years old with surveillance endoscopies/enteroscopies and an extensive exploratory laparotomy with complete enteroscopy resulting in resection of 33 jejunal polyps and subsequent small bowel resections. Over this time, he was found to have multiple adenomas in the duodenum, jejunum, major papilla, and ileal pouch with each endoscopy that occurred at ~3-month intervals with pathology including lesions ranging from low-grade dysplasia to invasive adenocarcinoma requiring surgical resection. Importantly, the proband and none of the family members with confirmed *EPCAM* deletion have developed tumors outside the GI tract. The patient undergoes routine surveillance for CMMRD-associated tumors^9^ without development of any brain tumors, hematologic malignancies or other cancers.

### EPCAM and MSH2 expression are tissue specific

**Fig. 1:**
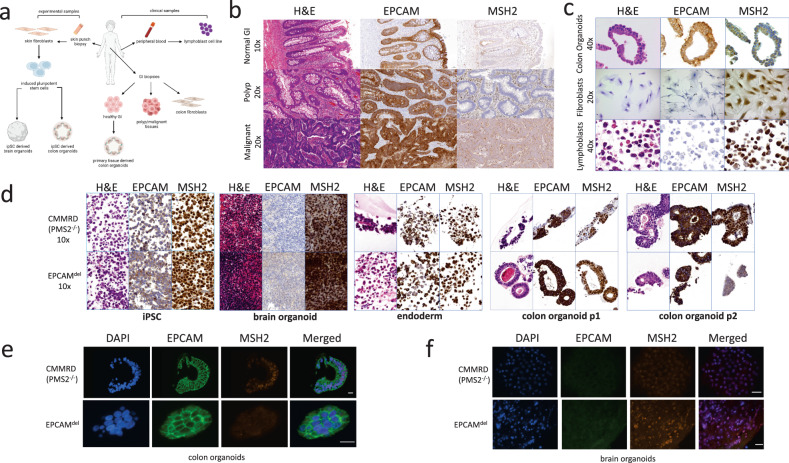
Immunohistochemistry and Immunofluorescence show differences in MSH2 and EPCAM staining in varied clinical samples from patient EPCAM^del^ and experimental models. **a** Details of clinical and experimental samples derived from patient EPCAM^del^. **b** IHC staining for H&E, EPCAM or MSH2 on normal (10×), polyp (20×) and malignant (20×) GI tissues taken from patient EPCAM^del^, showing EPCAM positivity and MSH2 negativity in all samples. **c** Immunohistochemistry staining for H&E, EPCAM or MSH2 on colon organoids (40×), fibroblasts (20×) and lymphoblasts (40×) from patient EPCAM^del^. Colon organoids show positive EPCAM staining and undetectable MSH2 staining. Fibroblasts and lymphoblasts show negative EPCAM staining and positive MSH2 staining **d** Immunohistochemistry staining for H&E, EPCAM or MSH2 on EPCAM^del^ iPSC, CMMRD PMS2^−/−^ iPSC, and iPSC-derived brain, endodermal and colon organoids (all 10×). iPSCs are negative for EPCAM and positive for MSH2. Brain organoids show negative EPCAM staining and positive MSH2. Some EPCAM positivity can be seen in the endodermal differentiation stage of colon organoid development and MSH2 staining remains mostly positive. Strong EPCAM staining can be seen at p1 of colon organoid differentiation and MSH2 staining is mostly retained in both CMMRD PMS2^-/-^ control samples and EPCAM^del^ samples. By p2 of differentiation, EPCAM staining remains strong in both, but EPCAM^del^ colon organoids exhibit complete loss of MSH2 staining, whereas CMMRD PMS2^−/−^ colon organoids maintain MSH2 expression. **e** Immunofluorescence staining for DAPI, EPCAM or MSH2 on EPCAM^del^ and CMMRD PMS2^−/−^ iPSC-derived colon organoids. Both CMMRD PMS2^−/−^ control and EPCAM^del^ show strong EPCAM staining, but colon organoids derived from EPCAM^del^ iPSC show loss of MSH2. The scale bars represent 50 µm. **f** Immunofluorescence staining for DAPI, EPCAM or MSH2 on EPCAM^del^ and CMMRD PMS2^−/−^ iPSC-derived brain organoids. Both CMMRD PMS2^−/−^ control and EPCAM^del^ iPSC-derived brain organoids show negative EPCAM staining, but positive MSH2 staining. The scale bars represent 50 µm.

To test our hypothesis of tissue-specific MMRD, several patient-derived tissues were sourced from patient EPCAM^del^ (summarized in Fig. 1a) including skin fibroblasts, healthy GI, adenomatous polyps resected during endoscopic surveillance and an adenocarcinoma. Additionally, lymphoblastoid cell lines and a colon organoid line were derived from blood and healthy GI tissue, respectively.

As in CMMRD, lack of MMR gene expression is associated with cancers in all organs^13^ but most commonly in lymphoid, brain and GI tissues, we first compared the relationship of EPCAM expression with MSH2 expression in our patient tissues. Immunohistochemistry (IHC) staining of non-malignant, pre-malignant (polyp) and malignant GI tissues (Fig. 1b) showed positive EPCAM staining and the associated loss of MSH2 in all tissues derived from the GI tract. Similar results were observed from primary tissue-derived colon organoids (Fig. 1c). In contrast to GI tissues, both lymphoblasts and fibroblasts revealed negative EPCAM staining and positive MSH2 staining (Fig. 1c).

Although individuals with CMMRD commonly develop brain tumors, patient EPCAM^del^ had never experienced malignancies in the brain and, therefore, no samples were available from neural tissue. To determine whether tissue-specific MMRD would only occur in tissues where *EPCAM* is expressed, sparing the neural system, we derived an induced-pluripotent stem cell (iPSC) line from skin fibroblasts isolated from the patient (summarized in Fig. 1a). From here, along with a control patient-derived *PMS2*^*−/−*^ CMMRD iPSC line previously established in our lab, we generated brain and colon organoid lines and subjected them to IHC analysis (Fig. 1d). As expected, both iPSC lines expressed MSH2 and had weak EPCAM positivity in some, but not all cells, representing non-specific staining. We then proceeded to create organ-specific organoids. We tested samples at 3 time points during colonic differentiation. The first sample was taken at the endodermal stage, 4 days post initiation. The second was the first passage of colon organoids (p1, day 16 post-differentiation), and the third was the second passage (p2, day 24 post-differentiation). At p1, colon organoids showed weak EPCAM positivity and positive MSH2 staining in both EPCAM^del^ and PMS2^−/−^ CMMRD control organoids. In the p2 colon organoids, the EPCAM^del^ samples exhibited strong EPCAM staining and undetectable MSH2, whereas the PMS2^-/-^ CMMRD control exhibited strong positivity for EPCAM and MSH2. This difference in EPCAM and MSH2 staining between p1 and p2 colon organoids suggests a progressive increase in *EPCAM* expression and loss of *MSH2* expression, over time, through differentiation to an epithelial cell type (Fig. 1d).

To test whether similar loss of expression of *MSH2* occurs in brain tissue, we then proceeded to generate brain organoids from control and EPCAM^del^ patient-derived iPSC lines. Samples were taken at week 7 of differentiation when brain organoids had developed to their terminal size. At week 7, both EPCAM^del^ and PMS2^−/−^ CMMRD control organoids showed no-to very weak EPCAM staining (which is likely to be non-specific) and strong MSH2 staining (Fig. 1d).

To further confirm the IHC results described above, immunofluorescence (IF) staining was conducted on iPSC-derived colon (Fig. 1e) and brain organoids (Fig. 1f), confirming that EPCAM^del^ iPSC yields EPCAM positive and MSH2-negative colon organoids, whereas PMS2^−/−^ CMMRD control organoids are EPCAM positive and retain MSH2. Both EPCAM^del^ and PMS2^−/−^ CMMRD control iPSC-derived brain organoids are negative for EPCAM and positive for MSH2.

### Causes and consequences of tissue-specific EPCAM expression

**Fig. 2:**
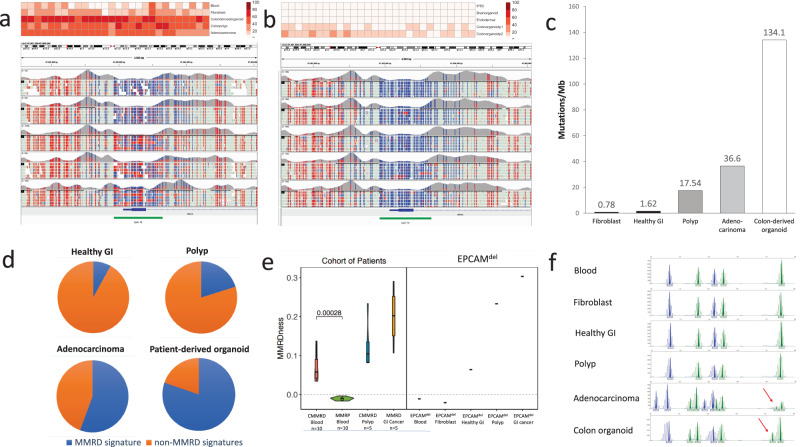
Epigenetic and genomic profiling of EPCAM^del^ samples show varied methylation, tumor mutation burden, mutation signatures, MMRD, and MSI across different tissue types. **a** CpG methylation analysis of the MSH2 promoter in EPCAM^del^ patient-derived tissues was substantially lower in blood and fibroblast samples than in GI-derived tissues. **b** In experimental tissues derived from patient EPCAM^del^, iPSC, brain organoid and endodermal tissue had low CpG methylation of the CpG promoter, whereas a stepwise increase in methylation was observed in colon organoids at passages 1 and 2. **c** Tumor mutation burden analysis of patient-derived samples normalized to blood showed a low TMB in fibroblast and healthy GI and significantly elevated TMB in polyp, adenocarcinoma and primary colon tissue derived cultured organoids. **d** Signature analysis of GI tissues from patient EPCAM^del^ showed healthy GI to have the lowest % of MMRD signature, with the % increasing for polyp and adenocarcinoma samples. The patient-derived colon organoid sample had the highest % contribution of MMRD signature. **e** LOGIC analysis confirmed MMR proficiency in EPCAM^del^ blood and fibroblasts and all GI-derived tissues had elevated MMRD scores, similar to scores found in MMRD polyps and cancers. **f** Promega panel MSI analysis indicated MSI stability in EPCAM^del^ patient-derived blood, fibroblast and healthy GI, slight increase MSI in colon polyp and high MSI in GI cancer and colon-derived organoids (indicated by red arrows).

Given the previously described role of DNA hypermethylation in *MSH2* promoter silencing due to *EPCAM* unterminated transcription, we utilized targeted methylation sequencing to map the DNA methylation status of the *MSH2* promoter in blood, fibroblast, colon-derived primary organoids, GI polyp and adenocarcinoma tissue. Methylation of the *MSH2* promoter was substantially lower in blood, lymphocytes and fibroblasts than in colon primary tissues and colon organoids cultured from primary GI tissue (Fig. 2a). A similar analysis was performed on patient-derived iPSC lines, as well as, iPSC-derived brain organoids, endodermal cells and two passages of iPSC-derived colon organoids (Fig. 2b). iPSC and brain organoids had a very low level of CpG methylation at the *MSH2* promoter, as did endodermal cells. Importantly, iPSC-derived colon organoids at passage 1 (p1) had a notable increase in CpG methylation, with this further increasing at passage 2 (p2) providing additional evidence of a stepwise increase in DNA methylation with further cell division after differentiation into an epithelial cell type.

To determine the consequences of *MSH2* promoter hypermethylation and consequent loss of gene expression on mutagenesis, whole exome sequencing (WES) was performed for these samples using matched blood as control (Fig. 2c). While fibroblasts had low Tumor Mutation Burden (TMB), an increase in TMB was observed between healthy GI tissue (1.62 mut/Mb), polyp (17.54 mut/Mb) and adenocarcinoma (36.6 mut/Mb). Furthermore, COSMIC signature analysis of these samples (Fig. 2d) revealed increases in the contribution of signature 6 which is the hallmark of MMRD-specific mutagenesis. Healthy GI had the lowest contribution (8.0%), polyp (21.2%), adenocarcinoma (55.6%), and the highest was the patient-derived organoid (79.8%). The colon-derived organoid sample had both an extremely high TMB (134.1mut/Mb), as well as, the highest signature 6 contribution likely due to the high number of cell divisions and the clonal nature of the intestinal crypt cells isolated from the patient and organoid growth in cell culture.

To further quantify the degree of MMRD in patient cells, we used the recently developed Low-pass Genomic Instability Characterization (LOGIC) assay, which can quantify the degree of MMRD through genome-wide microsatellite instability signatures^7,14^. MMRDness scores (Fig. 2e) were negative (<0.0) for EPCAM^del^ blood and fibroblasts indicating MMR proficiency. In contrast, all GI tissues had high MMRD scores with increased level between normal to malignant tissues comparable to CMMRD tissues of the same type (Fig. 2e).

These results were further confirmed by using targeted Promega panel MSI sequencing (Fig. 2f) commonly used in the diagnosis of MMRD. The blood, fibroblast and healthy GI tissue were MSI stable. In contrast, the polyp showed a slight increase in MSI and both the colon-derived organoid and GI cancer from the patient showed high levels of MSI.

## Discussion

Here we use developmental biology and functional genomic tools to demonstrate that *EPCAM* deletion results in MMRD that is limited to tissues in which *EPCAM* is expressed. This unique tissue-specific germline predisposition has both biological and clinical implications for the role of promoter hypermethylation in MMRD and the management of individuals with germline biallelic *EPCAM* deletion.

As CMMRD is a multiorgan cancer predisposition syndrome, the concept of tissue-specific CMMRD remains unexplored. It is challenging to sample normal tissue from many organs unaffected by tumors, but the use of iPSC and organoid technologies can help in determining whether an underlying mechanism such as MMRD exists in such tissues. Most cancers affecting CMMRD individuals include brain, hematological, and GI tissues. To model these tissues, we chose to use specific cell types (lymphoblasts, brain and colon organoids) and assess the potential risk of carcinogenesis in these major organs, concluding that lymphoblast and brain tissues (representing ectodermal and mesodermal origins) are MMR-proficient in EPCAM deleted tissues and should not be at increased risk for malignancy. In addition, the fibroblast analysis showing proficient MSH2 suggests a low chance of sarcomas and cancers in tissues of mesenchymal origin among individuals with homozygous *EPCAM* deletion. In contrast, colon tissues which represent endodermal-originated epithelial cells are MSH2 deficient and reveal extreme mutagenesis leading to an increased risk of malignancy.

Organoids are commonly used for genetic or drug screens and to model organ development^15^. The use of organoids to determine the mechanisms which govern cancer development and lack of mutagenesis during normal organ development is, to our knowledge, quite unique.

The combination of iPSC-derived organoids and functional genomic analysis, which provides a quantifiable measure of MMRD, allowed us to provide insight into the relationship between *EPCAM* expression and the emergence of MMRD during development. In addition, this technology can be used to further elucidate the mechanisms of DNA mismatch repair deficiency via ongoing hypermethylation of the *MSH2* promoter during GI tissue development. As hypermethylation occurs somatically in *MLH1* causing MMRD cancers^16,17^, these novel tools can be used to better understand the mechanisms leading to such a process during carcinogenesis. This method of mimicking tissue development and following its functional consequences can be used as a proof of principle for other DNA damage repair defects and cancer predisposition conditions.

Finally, surveillance recommendations for individuals with CMMRD include a brain MRI every 6 months beginning at birth, complete blood count and abdominal ultrasound every 6 months beginning at age 1, and yearly whole-body MRI, upper and lower endoscopy and capsule endoscopy beginning at age 4–6 years old^9^. Hence our results suggest that patients with homozygous *EPCAM* mutations may not need surveillance on tissues which do not have *EPCAM* expression as the data presented here suggest that only these specific tissues are at an increased risk of malignancy. Since the age of cancer onset in biallelic EPCAM deletion carriers is extremely early, GI surveillance may be recommended at even younger age as for CMMRD patients harboring germline mutations in other MMR genes.

Further exploration of the exact tissues affected in LS patients harboring heterozygous *EPCAM* deletion may require further investigation.

In summary, this proof of principle study reveals the use of tissue engineering and functional genomic tools to define processes governing tumorigenesis during development and to determine both the risk of cancer and specific interventions in a tissue-specific manner. These will reduce the need for over-testing including complex imaging and invasive tests for these young children.

## Methods

### Generation of skin fibroblast line from skin punch biopsy

A skin punch biopsy sample was cultured in high-glucose DMEM-based media supplemented with 15% FBS, 1x L-glutamine, 1x, MEM-NEAA, sodium pyruvate, 1x pen/strep, 1x gentamycin, 1x amphotericin for up to two weeks until fibroblast cultures were established. These cells were then transferred to high-glucose DMEM media supplemented with 10% FBS, 1x L-glutamine, 1x sodium pyruvate and 1x non-essential amino acids without antibiotics. Cells were split at a 1:6 ratio when confluent in 10 cm dishes and frozen down using culture media with 40% FBS and 10% DMSO.

### Generation of iPSC from skin fibroblast line

Reprogramming of patient-derived skin fibroblasts was done by the Centre for Commercialization of Regenerative Medicine (CCRM), Toronto, Canada. Briefly, viral reprogramming was done using SeV Vectors encoding Oct3/4, Sox2, Klf4 and c-Myc from the CytoTune™-iPS 2.0 Sendai Reprogramming Kit (Thermo Fisher) according to manufacturer’s protocol. 24 h post-infection the virus is washed off the cells and resuspended in fresh media. 48 h after viral transduction, reprogrammed cells were plated at different densities on Matrigel-coated six-well plates in SFII. ReproTeSR (1 mL) was added to the wells on days 3 and 5 after reprogramming. 7 days after reprogramming the media is changed daily with ReproTeSR. Once colonies are of adequate size and morphology to pick, cells are fed with E8 media. 24 h after switching to E8 media, individual colonies are picked and plated clonally. Colonies were assessed for quality, pluripotency, germ layer differentiation, karyotype, viability as detailed previously^18^. All cell culture reagents from STEMCell Technologies if not specified. iPSC lines were cultured in mTESR media on Matrigel (Corning Life Sciences) coated six-well plates. Colonies were passaged when ~50% confluent by the surface area of the well and removed with ReLeSR™ reagent. Colonies were fed every 2 days with fresh media, irrespective of confluence. Differentiated cells were occasionally removed mechanically with a pipette tip as well as using ReLeSR™ reagent to ensure pluripotency of cultures.

### Generation of LCL line from patient blood sample

Mononuclear cells were separated from peripheral blood samples via Ficoll-Plaque extraction (company). Cells were washed and suspended in filtered supernatant from B95-8, an EBV-producing Marmoset lymphoblast line. Cells were left in a 37 °C, 5% CO_2_ incubator for a minimum of 1 week and monitored for signs of growth via media color change. Once 70–80% confluent, cells were split 1:3 and/or biobanked in liquid nitrogen for long-term storage.

### Generation of brain and colon organoids

Human cerebral organoids were generated from iPSC using the STEMdiff™ Cerebral Organoid Kit (Cat # 08570) following the manufacturer’s protocol. Cerebral organoids were harvested at week 7 post-seeding and either flash-frozen for DNA extraction and sequencing or fixed and embedded for IHC/IF analysis. Colon organoids were generated from primary patient colon tissue or iPSC. Primary patient colon tissue was used to harvest intestinal crypts before seeding for culture using IntestiCult™ Organoid Growth Medium (Cat #06010) using the manufacturer’s protocol. Intestinal organoids were generated from iPSC using the STEMdiff™ Intestinal Organoid Kit (Cat #05140) following the manufacturer’s protocol. All kits were supplied by StemCell Technologies, Vancouver, Canada.

### Immunohistochemistry analysis of iPSC, cerebral, and colon organoids

iPSC or endodermal layers were removed from Matrigel-coated plates using ReLeaSR, washed in PBS and pelleted, fixing in formalin for 30 min before resuspending in PBS and paraffin-embedded. Colon organoids were removed from Matrigel using GCDR, washed with PBS and fixed in formalin for 45 min before pelleting and resuspending in PBS. Embedding of these tissues was done by Pathology Research Program Laboratory (PRP), UHN, Toronto, Canada. Cerebral organoids were fixed in 70% ethanol for 24 h before being paraffin embedded by Pathology Core Centre for Modeling Human Disease at The Centre for Phenogenomics, Toronto, Canada. All fixed samples were cut to 10 μM (cerebral organoids) or 4 μM (iPSC, endodermal layer, colon organoids) and mounted on slides before being stained with H&E and analyzed for EPCAM (Abcam, ab46714, HEA125, prediluted used at stock concentration, overnight staining) and MSH2 (Pharmingen 556349, clone G219-1129, 1/500, 1 h staining) and the Mach 4 universal-HRP polymer kit.

### Immunofluorescence analysis of iPSC-derived tissues

Slides were prepared as above for IHC staining. All slides with tissue preparations were incubated at 65 °C for 10 min to melt paraffin. Slides were washed in Xylene three times for 5 min each. Slides were rehydrated sequentially in 100% (2×), 95% (2×), 70% (1×) of ethanol for 3 min each, then washed with water for 3 min and 1% Tween for 1 min. Slides were incubated in Citrate buffer at 95 °C for 30 min to unmask antigens and washed again with H_2_O. Slides were treated with blocking solution at 37 °C for 30 min. Primary antibodies were added at indicated dilutions (Supplementary Fig. 1) and incubated overnight at 4 °C. The following day, slides were washed three times with blocking solution before incubating with secondary antibodies (Supplementary Fig. 1) at a 1:500 dilution for 1 h at room temperature. Slides were stained with DAPI and washed with PBS before adding final mounting solution.

Primary antibodies used were; Rabbit anti-EPCAM at 1:1000 dilution, Abcam, Cat# ab71916, Mouse anti-MSH2 at 1:100, from ThermoFisher Scientific, Cat# 33-7900, Sheep anti-Ki67, at 1:100, R&D Systems, Cat# AF7617. Secondary antibodies used were: Anti-rabbit A488, at 1:500, Anti-mouse Cy3, at 1:500 and Anti-sheep A647 at 1:500, all from Jackson ImmunoResearch.

### Analysis of methylation

Library preparation, sequencing, and methylation analysis were carried out as described previously^11^.

### Analysis of tumor mutation burden (TMB)

WES was performed at The Centre for Applied Genomics (TCAG), SickKids, using SureSelect Agilent All Exon v5 kit, followed by sequencing (150×) on Illumina HiSeq 2500. The software bcl2fastq2 v2.17 was used to generate raw fastq files. Alignment to the hg38 reference genome, followed by pre-processing and QC was adapted from the GATK standard pipeline, using BWA-MEM 0.7.12 (alignment), BAMQC, Picard 2.6.0 (QC). Somatic variant calling was done post-alignment, using processed bam files from tumor and matched normal samples, to call both single nucleotide variants (SNVs) and insertion deletion (indel) variants. A consensus vcf file of shared variants across 2 or more variant callers (Mutect v1.1.5, GATK v3.6/Mutect2, Strelka v1.0.14, and Varscan2 Somatic v2.4.2) was generated for SNVs and indels separately, using VCFtools 0.1.15, and these vcfs were annotated using VEP v83. The tumor mutation burden (SNVs per megabase) from WES data was calculated by counting total number of somatic SNVs divided by total number of callable bases in megabases (~50 Mb). More detailed methods are described in ref. 19.

### Signature analysis

Signature analysis was done on somatic variants using sigminer (https://github.com/ShixiangWang/sigminer). We performed signature analysis for each sample by refitting to both COSMIC signature V3.2 and V2.0 using the MutationalPatterns R Package (v3.4.a)^20^.

### Analysis of MSI—LOGIC

Samples were sequenced at 1X coverage using the NovaSeq6000 sequencer (Illumina). Microsatellite indels were called using an established algorithm^21^ and MMRDness score calculations were described in a previous report^7^. Briefly, the number of microsatellite deletions of 1 bp in loci of 10–15 bp was divided by the total number of genomic loci at each length, which was then averaged and a logarithmic transformation was applied to calculate the score. A final scalar transformation of +1.1 was added to normalize the MMRDness score threshold at 0.

### Analysis of MSI—Promega panel

DNA extracted from patient non-malignant, polyp and tumor samples as well as patient-derived organoids were quantified with Nanodrop (Thermo Fisher Scientific) and amplified with Platinum Multiplex PCR Master Mix (Thermo Fisher Scientific) and MSI 10X Primer Pair Mix (Promega) in a Veriti 96-Well Thermal Cycler, using the manufacturer’s recommendations for PCR cycling conditions. The primer mix targets a panel of five mononucleotide loci: BAT-25, BAT-26, NR-21, NR-24, and MONO-27. After amplification, the products were run in a 3130 Genetic Analyzer for fluorescent capillary electrophoresis. Electrophoretograms were visualized using Peak Scanner Software (v1.0, Thermo Fisher Scientific), and the highest peaks that were flanked by lower peaks were selected to be the representative alleles for each of the five loci in the panel. Each sample was compared by its allelic length. Samples were considered MSI-High if two or more loci were unstable (≥3 bp shift from the normal allele), MSI-Low if one locus was unstable, and MS-Stable if all five loci were stable (≤2 bp shift from the normal allele)^22^.

### Consent for study participation

This study has complied with all relevant ethical regulations, including the principles outlined in the Declaration of Helsinki. The study protocol received approval from SickKids Research Ethics Board at The Hospital for Sick Children. All participants provided informed written consent allowing for their data and samples to be included in this study and published.

### Reporting summary

Further information on research design is available in the Nature Research Reporting Summary linked to this article.

### Supplementary information

Supplementary Figure 1

REPORTING SUMMARY

## Data Availability

All data can be obtained directly from the International Replication Repair Deficiency Consortium (IRRDC). Please contact the consortium (replication.repair@sickkids.ca) with the details of your request and a short description of your project. Sequencing data from this study has not been deposited into online repositories due to patient consent requirements, which require that data be released in aggregate with other participants. However, interested researchers may contact the IRRDC or corresponding author to access the data through collaboration or data access agreements.

## Code availability

No custom code was developed for this paper. Software, versions and associated parameters used can be found in the methods section. Microsatellite indels were called using an established algorithm and MMRDness score calculations were described in Chung, 2021. Requests to use this algorithm can be submitted directly to the IRRDC (replication.repair@sickkids.ca).
