# Supplementary material for: Biallelic EPCAM deletions induce tissue-specific DNA repair deficiency and cancer predisposition
Source: NPJ Precis Oncol. 2024 Mar 11;8:69. doi: 10.1038/s41698-024-00537-6 (PMC10928233; doi:10.1038/s41698-024-00537-6)
Supplement: Supplementary file 1 — Supplementary Figure 1 [file 41698_2024_537_MOESM1_ESM.pdf]

a

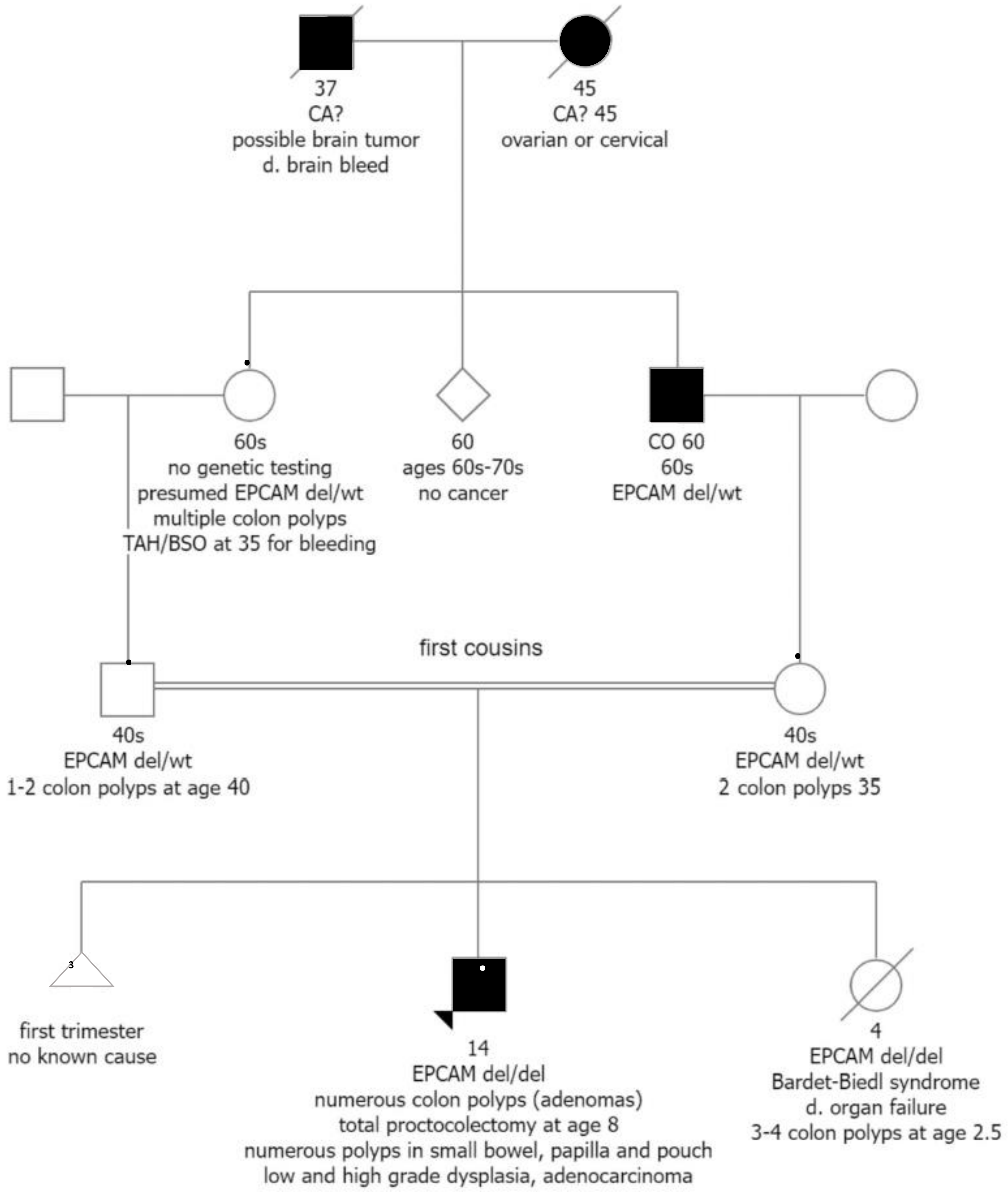

b

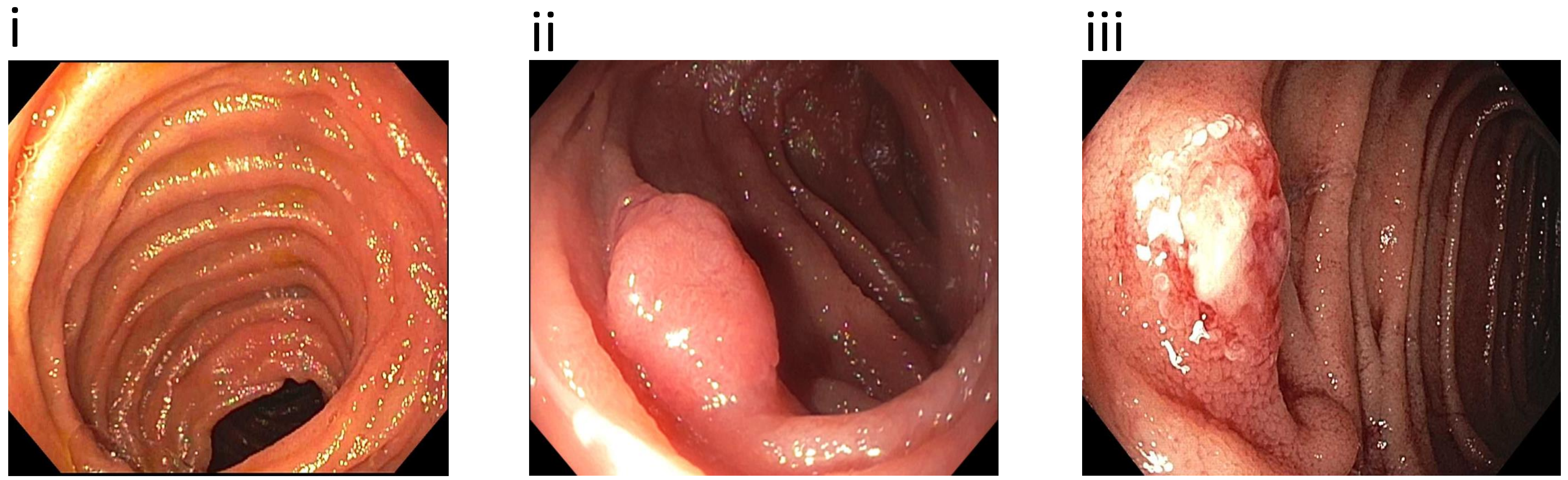

Normal jejunum (healthy GI)

Medium sessile jejunal adenoma (polyp)

Jejunal adenocarcinoma

Supplementary Figure 1:. a: Pedigree of the proband and extended family. Proband is marked by an arrow. **b:** Scope pictures showing healthy GI tissue (normal jejunum), polyp (medium sessile jejunal adenoma) and jejunal adenocarcinoma.
