# Supplementary material for: Biallelic EPCAM deletions induce tissue-specific DNA repair deficiency and cancer predisposition
Source: NPJ Precis Oncol. 2024 Mar 11;8:69. doi: 10.1038/s41698-024-00537-6 (PMC10928233; doi:10.1038/s41698-024-00537-6)
Supplement: Supplementary file 2 — REPORTING SUMMARY [file 41698_2024_537_MOESM2_ESM.pdf]

nature portfolio

Corresponding author(s): Dr. Uri Tabori
Last updated by author(s): Dec 1, 2023

# Reporting Summary

Nature Portfolio wishes to improve the reproducibility of the work that we publish. This form provides structure for consistency and transparency in reporting. For further information on Nature Portfolio policies, see our Editorial Policies and the Editorial Policy Checklist.

## Statistics

For all statistical analyses, confirm that the following items are present in the figure legend, table legend, main text, or Methods section.

| n/a | Confirmed | |
|---|---|---|
| ☐ | ☒ | The exact sample size (*n*) for each experimental group/condition, given as a discrete number and unit of measurement |
| ☐ | ☒ | A statement on whether measurements were taken from distinct samples or whether the same sample was measured repeatedly |
| ☐ | ☒ | The statistical test(s) used AND whether they are one- or two-sided<br>*Only common tests should be described solely by name; describe more complex techniques in the Methods section.* |
| ☒ | ☐ | A description of all covariates tested |
| ☒ | ☐ | A description of any assumptions or corrections, such as tests of normality and adjustment for multiple comparisons |
| ☐ | ☒ | A full description of the statistical parameters including central tendency (e.g. means) or other basic estimates (e.g. regression coefficient) AND variation (e.g. standard deviation) or associated estimates of uncertainty (e.g. confidence intervals) |
| ☒ | ☐ | For null hypothesis testing, the test statistic (e.g. *F*, *t*, *r*) with confidence intervals, effect sizes, degrees of freedom and *P* value noted<br>*Give P values as exact values whenever suitable.* |
| ☒ | ☐ | For Bayesian analysis, information on the choice of priors and Markov chain Monte Carlo settings |
| ☒ | ☐ | For hierarchical and complex designs, identification of the appropriate level for tests and full reporting of outcomes |
| ☒ | ☐ | Estimates of effect sizes (e.g. Cohen's *d*, Pearson's *r*), indicating how they were calculated |

*Our web collection on statistics for biologists contains articles on many of the points above.*

## Software and code

Policy information about availability of computer code

| | |
|---|---|
| Data collection | No software was used for data collection |
| Data analysis | The software bcl2fastq2 v2.17 was used to generate raw fastq files. Alignment to the hg38 reference genome, followed by pre-processing and QC was adapted from the GATK standard pipeline, using BWA-MEM 0.7.12 (alignment), BAMQC, Picard 2.6.0 (QC). Somatic variant calling was done post-alignment, using processed bam files from tumour and matched normal samples, to call both single nucleotide variants (SNVs) and insertion deletion (indel) variants. A consensus vcf file of shared variants across 2 or more of variant callers (Mutect v1.1.5, GATK v3.6/ Mutect2, Strelka v1.0.14, and Varscan2 Somatic v2.4.2) was generated for SNVs and indels separately, using VCFtools 0.1.15, and these vcfs were annotated using VEP v83. Signature analysis was done on somatic variants using sigminer (https://github.com/ShixiangWang/sigminer). We performed signature analysis for each sample by refitting to both COSMIC signature V3.2 and V2.0 using the MutationalPatterns R Package (v3.4.a) Microsatellite indels were called using an established algorithm and MMRDness score calculations were described in Chung, 2021. Briefly, the number of microsatellite deletions of 1bp in loci of 10-15bp were divided by the total number of genomic loci at each length, which was then averaged and a logarithmic transformation was applied to calculate the score. Electrophoretograms for MSI analysis were visualized using Peak Scanner Software (v1.0, Thermo Fisher Scientific), and the highest peaks that were flanked by lower peaks were selected to be the representative alleles for each of the five loci in the panel. |

For manuscripts utilizing custom algorithms or software that are central to the research but not yet described in published literature, software must be made available to editors and reviewers. We strongly encourage code deposition in a community repository (e.g. GitHub). See the Nature Portfolio guidelines for submitting code & software for further information.

## Data

Policy information about availability of data

All manuscripts must include a data availability statement. This statement should provide the following information, where applicable:

- Accession codes, unique identifiers, or web links for publicly available datasets
- A description of any restrictions on data availability
- For clinical datasets or third party data, please ensure that the statement adheres to our policy

Data can be obtained directly from the International Replication Repair Deficiency Consortium. Please contact the consortium (replication.repair@sickkids.ca) directly with the details of your request, and a short description of your project.

## Research involving human participants, their data, or biological material

Policy information about studies with human participants or human data. See also policy information about sex, gender (identity/presentation), and sexual orientation and race, ethnicity and racism.

| | |
|---|---|
| Reporting on sex and gender | As the EPCAM and MSH2 genes are not linked to the sex chromosomes, all findings should apply to both sexes. As this was a n=1 study, sex and/or gender were not considered in the study design. Gender and sex were determined by self-reporting, as well as the methods used. |
| Reporting on race, ethnicity, or other socially relevant groupings | There are no groupings in this manuscript related to race, ethnicity or other socially relevant groupings. |
| Population characteristics | There are no covariate-related population characteristics in this manuscript |
| Recruitment | The patient was referred to the Replication Repair Deficiency Biobank at the Hospital for Sick Children by their treating physician following identification of their condition. The patient was consented by the coordinator for this study, and all samples were collected under the biobank REB. |
| Ethics oversight | The Hospital for Sick Children - Replication Repair Deficiency Biobank (REB#1000048813) |

Note that full information on the approval of the study protocol must also be provided in the manuscript.

# Field-specific reporting

Please select the one below that is the best fit for your research. If you are not sure, read the appropriate sections before making your selection.

☒ Life sciences ☐ Behavioural & social sciences ☐ Ecological, evolutionary & environmental sciences

For a reference copy of the document with all sections, see nature.com/documents/nr-reporting-summary-flat.pdf

# Life sciences study design

All studies must disclose on these points even when the disclosure is negative.

| | |
|---|---|
| Sample size | The sample size for this manuscript was one of convenience. This is a case study highlighting a single patient identified with a rare genetic condition. |
| Data exclusions | No data was excluded from the manuscript |
| Replication | Each of the biological experiments was repeated 3 times as required and for genomic analysis, 2 methods at least were used (For example methylation analysis). |
| Randomization | Randomization was not relevant to this study, as samples were collected from a single individual. Samples were allocated to groups based on their tissue of origin. |
| Blinding | As this was a case study, blinding was not relevant to this study |

# Reporting for specific materials, systems and methods

We require information from authors about some types of materials, experimental systems and methods used in many studies. Here, indicate whether each material, system or method listed is relevant to your study. If you are not sure if a list item applies to your research, read the appropriate section before selecting a response.

## Materials & experimental systems

| n/a | Involved in the study | |
|---|---|---|
| ☐ | ☒ | Antibodies |
| ☐ | ☒ | Eukaryotic cell lines |
| ☒ | ☐ | Palaeontology and archaeology |
| ☒ | ☐ | Animals and other organisms |
| ☒ | ☐ | Clinical data |
| ☒ | ☐ | Dual use research of concern |
| ☒ | ☐ | Plants |

## Methods

| n/a | Involved in the study | |
|---|---|---|
| ☒ | ☐ | ChIP-seq |
| ☒ | ☐ | Flow cytometry |
| ☒ | ☐ | MRI-based neuroimaging |

# Antibodies

Antibodies used

For IHC analysis analysis, samples were stained using the EPCAM (Abcam, ab46714) and MSH2 (Pharmingen 556349) anti-bodies. For the immunofluorescence analysis, Rabbit anti-EPCAM Abcam, Cat# ab71916, Mouse anti-MSH2, Cat# 33-7900, and Sheep anti-Ki67 R&D Systems, Cat# AF7617 were used.

Validation

All primary antibodies used in this study were validated by manufacturers and validation statement for each antibody is provided on the manufacture's website.

Abcam, ab46714
Mouse Monoclonal EPCAM antibody. Validated in ICC/IF and tested in Human samples. Cited in 2 publications.
https://www.abcam.com/products/primary-antibodies/epcam-antibody-hea125-prediluted-ab46714.html

Pharmingen 556349
Cited 8 times
https://www.bdbiosciences.com/en-us/products/reagents/microscopy-imaging-reagents/immunohistochemistry-reagents/Purified-Mouse-Anti-Human-MSH-2.556349

Cat#ab71916
Rabbit Polyclonal EPCAM antibody. Validated in WB, IHC-P, ICC/IF and tested in Mouse, Rat, Human samples. Cited in 115 publications.
https://www.abcam.com/products/primary-antibodies/epcam-antibody-ab71916.html

Cat#33-7900
Advanced Verification -IP-mass spectrometry
3 Published Figures
41 References
https://www.thermofisher.com/antibody/product/MSH2-Antibody-clone-FE11-Monoclonal/33-7900

Cat# AF7617.
7 verified figures
5 References
https://www.rndsystems.com/products/human-ki67-mki67-antibody-1297a_mab7617

# Eukaryotic cell lines

Policy information about cell lines and Sex and Gender in Research

Cell line source(s)

Lymphoblastoid cell lines and a colon organoid line were derived from blood and healthy GI tissue, respectively. Fibroblasts were derived from a skin biopsy. All cell lines are male

Authentication

As these are patient derived cells, validation was done by specific mutational anlysis of the germline condition in the cells.

Mycoplasma contamination

All cell lines were tested for Mycoplasma contamination

Commonly misidentified lines
(See ICLAC register)

No commonly mis-identified lines were used

## Plants

| | |
|---|---|
| Seed stocks | N/A |
| Novel plant genotypes | N/A |
| Authentication | N/A |
